# The association between bone turnover markers and microvascular complications of type 2 diabetes

**DOI:** 10.1186/s40200-016-0274-2

**Published:** 2016-11-07

**Authors:** Zhila Maghbooli, Parisa Shabani, Sattar Gorgani-Firuzjaee, Arash Hossein-nezhad

**Affiliations:** 1 Endocrinology and Metabolism Clinical Sciences Institute of Tehran University of Medical Sciences, Tehran, Iran; 2 Clinical Biochemistry Department, Faculty of Medicine, Tehran University of Medical Sciences, Tehran, Iran; 3 Department of Medical Laboratory Sciences, School of Allied Health Medicine, AJA University of Medical Sciences, Tehran, Iran; 4 Osteoporosis Research Center, Endocrinology and Metabolism Clinical Sciences Institute, Tehran University of Medical Sciences, Postal address; EMRI, 5th floor, Shariati Hospital, North Karegar Avenue, P.O Box: 1411413137, Tehran, Iran

**Keywords:** Type 2 Diabetes, Retinopathy, Nephropathy, Bone turnover markers, PTH, eGFR, BUN

## Abstract

**Background:**

Global epidemic of diabetes is a serious health care concern because of its complications and consequently reduced life expectancy and increased morbidity. However, the bone turnover and thus bone health may be affected or even compromised by diabetes and its complications. The aim of this study was to assess whether bone turnover markers are associated with diabetes micro-vascular complications.

**Methods:**

A total of 204 type 2 diabetes patients (104 patients with diabetic micro-vascular complications (retinopathy and/or nephropathy) as a case group and 100 patients without retinopathy and/or nephropathy) as a control group were recruited in this case–control study. The biochemical and metabolic parameters and bone turnover markers were assessed in all patients.

**Results:**

Our findings showed serum levels of osteocalcin (OC) (*p =* 0.0001) and, carboxy-terminal collagen crosslinks (CTX) (*p =* 0.006) were higher in diabetic patients with both diabetic retinopathy and nephropathy compared with control group. However, there was no significant difference in serum levels of procollagen I aminoterminal propeptide (P1NP) between diabetic patients with diabetic retinopathy (DR) and/or diabetic nephropathy (DN) compared with control.

In diabetes patients with complications, there were significant negative correlation between OC and CTX with estimated-glomerular filtration rate (e-GFR) and also positive correlation between each bone marker (OC and CTX) and PTH levels (*p* = 0.0001) and BUN (*p* = 0.0001).

In a general linear model, after adjusting for age, sex and BMI, and microvascular complications, there was not any significant association between three bone turnover markers and metabolic markers including fasting glucose, insulin, and lipid profile. Among kidney markers, there were significant positive associations between serum levels of CTX and OC with BUN (*p* < 0.05) as well as PTH (*p* < 0.0001).

**Conclusions:**

Our data suggest the possible role of PTH and BUN levels in modulating bone turnover markers in diabetic patients.

## Background

Osteoporosis, known as the silent disease, has become an important public health issue and highly prevalent condition affecting hundreds of millions of people around the world. It is characterized by reduced bone mass and altered microarchitecture [1]. Aging is the most significant risk factor for osteoporosis. Considering the global trend in population aging, skeletal health is an important consideration for older adults in general and health priority in worldwide [2].

Type 2 diabetes (T2D) is a common age-related disorder that imposes a growing medical and economic burden in ageing populations. Growing evidence suggests that patients with type 2 diabetes are at increased risk of bone fragility [3, 4]. Several studies have demonstrated increased risk of fracture in diabetic patients in spite of normal or even higher bone mineral density (BMD). Longer disease duration, poor glycemic control and particularly diabetic complications could increase the fracture risk in diabetic patients [3]. A few studies have addressed the association of chronic complications of T2D with reduced BMD. They have reported decreased BMD in patients with neuropathy [4, 5], retinopathy [6] and also in patients with early-stage of diabetic chronic kidney disease [7]. Recent studies have demonstrated that diabetes may adversely influence the bone quality through the regulation of bone cells [8].

Bone turnover markers (BTMs) are peptides secreted by bone cells and reflect both bone formation (osteocalcin, procollagen I aminoterminal propeptide) and bone resorption (carboxy-terminal collagen crosslinks), and consequently indicate bone remodeling status; which is considered the major mechanism underlying osteoporosis [9]. BTMs have been proposed to be independent predictor factors for osteoporosis [10–13] and osteoporotic fractures [14–16]. It has been suggested that osteocalcin (OC) production is diminished by negative regulation of osteoblasts in diabetes condition [8, 17]. Also, bone resorption markers such as carboxy-terminal collagen crosslinks (CTX) has been proposed as a predictive factor of bone fracture in diabetes patients [18].

Several studies have implicated that BTMs are more sensitive than BMD in estimating fracture risk in patients with diabetes owing to linkage between BTMs and glucose metabolism [19, 20].

However, few studies address the association between BTMs and diabetic complications. We hypothesize that circulating levels of BTMs can be helpful for management of osteoporosis and prevention of bone fracture in presence of diabetes complications.

The aim of this study was to assess whether bone turnover markers were associated with diabetes micro-vascular complications. Moreover, the possible association between bone turnover markers and prognostic factors of diabetes were concomitantly evaluated.

## Methods

### Study population

Between July 2012 and September 2013, 204 adults with type 2 diabetes mellitus,104 patients with retinopathy and or nephropathy and 100 patients without retinopathy and nephropathy were recruited in this case–control study from a referral diabetes clinic that is affiliated with the Tehran University of Medical Sciences. The study was approved by the Ethics Committee of the Endocrinology and Metabolism Research Institute. A comprehensive questionnaire was used and written informed consent was obtained from all participants.

The inclusion and exclusion criteria, retinopathy and nephropathy diagnosis criteria of the study population have been described previously [21–23].

The diagnosis of type 2 diabetes was carried out and/or confirmed following the American Diabetes Association criteria (ADA) [24], which includes a fasting blood glucose ≥126 mg/dL on two separate occasions, random (non-fasting) blood glucose ≥200 mg/dL on two separate occasions or a blood glucose >200 mg/dL at 2 h during a standard oral glucose tolerance test.

### Retinopathy and nephropathy definition

All patients were type 2 diabetes without any history of cancer or chronic disorders. The diagnosis of retinopathy was carried out according to the American Academy of Ophthalmology recommendations [25]. According to previously described definitions, a urine albumin-to-creatinine ratio was determined from a random urine collection of all patients and classified as normal (urine microalbumin: creatinine ratio ≤ 30 μg/mg), micro albuminuria (urine microalbumin: creatinine ratio >30 μg/mg and ≤ 299 μg/mg) and macro albuminuria (urine microalbumin: creatinine ratio ≥ 300 μg/mg) at least on two separate occasions.

The control subjects were diabetic patients with urinary albumin excretion values within the normal range, without retinopathy.

### Diabetes risk factor definition

Diabetes risk factors were defined based on ADA criteria [24]. Hypertension was defined in subjects with a BP ≥ 140/90 mm Hg or current use of high blood pressure medications. Dyslipidemia was defined as TG >250 mg/dL and/or HDL < 35 mg/dL or using lipid-lowering medications. Glycemic control was categorized into poor and good glycemic control based on HbA1c ≥ 7 % or HbA1c < 7 %, respectively. Obesity was classified based on BMI > 30 kg/m^2^.

### Data collection and measurements

Demographic and clinical characteristics including sex, age, age at diabetes diagnosis, diabetes duration, cigarette smoking status, and current use of medications were obtained by a questionnaire. Height and weight were measured using standard anthropometric techniques. Body mass index was calculated as body weight (kg)/(height (m^2^). Blood pressure was measured twice after a 10 min seated rest. Blood samples were collected after an overnight fasting. The sera were kept at −80 °C until analysis. Serum biochemical parameters including glucose, total cholesterol (TC), high-density lipoprotein (HDL), low-density lipoprotein (LDL), triglyceride (TG), blood urea nitrogen (BUN), uric acid, and creatinine (Cr), aspartate aminotransferase (AST), alanine aminotransferase (ALT) and urine microalbumin and creatinine levels were measured by enzymatic colorimetric assay [Pars-Asmun kits, Iran] using an auto analyzer [Hitachi 902, Japan]. The serum insulin level was measured by an immunoenzymometric assay [Monobind Inc., USA]. The intra- and inter-assay coefficients of variation (CVs) for insulin were 5.9 % and 9.2 %, respectively. Glycated hemoglobin (HbA1c) level was assessed using ion exchange chromatography with a DS5 set [DREW, United Kingdom].

Intact parathyroid hormone (PTH), OC and CTX were measured using electrochemiluminescence assay (Roche). In electrochemiluminescence assay for OC a large N-terminal midfragment was recognized in addition to the intact molecule.

Serum 25(OH)D was measured by radioimmunoassay (RIA) using a Biosource kit (Biosource Europe SA, Belgium); intra- and inter-assay coefficients of variation (CV) were 5.2 % and 7.5 %, respectively. P1NP was measured by quantitative sandwich enzyme immunoassay technique (intra- and inter-assay CV was <8 % and <10 %) (Cusabio).

Estimating GFR was calculated based on CKD EPI Equation; for Cr > 61.9 μmol/l [if female]: GFR = 144 × (S_cr_/61.9)^-1.209^ × (0.993)^Age^, for Cr > 79.6 μmol/l [if male] GFR = 141 × (S_cr_/79.6)^-1.209^ × (0.993)^Age^. In our population study, serum Cr levels were over 61.9 μmol/l in women and over 79.6 μmol/l in men.

### Statistical analysis

Statistical analysis was performed using SPSS software (version 16). As certain data, including fasting serum blood glucose, LDL, TC, TG, PTH, insulin, Cr, uric-acid, OC, CTX, and P1NP levels did not have normal distributions in case or control group; log transformation was applied to correct their normality distribution.

Student’s t-test was used to compare the differences in serum levels of bone turnover markers in diabetic patients with and without micro-vascular complications. ANOVA and post Hoc tests were applied to examine the difference between bone turnover markers in patients with diabetic retinopathy (DR), diabetic nephropathy (DN), and both DR/DN. A Pearson correlation was used to determine correlation between bone turnover markers and metabolic markers. A general linear model was used to determine an independent association between bone turnover markers and diabetes micro-vascular complications.

Numerical variables were reported as the mean ± standard error, and categorical variables were presented as percentages. Two-tailed *p-*values less than 0.05 were considered significant.

## Results

A total of 204 diabetic patients were enrolled in this study that included 104 patients with DR and/or DN (case group) and 100 patients without DR and/or DN (control group). The baseline characteristics of diabetic patients with and without the complications are shown in Table 1. Case group was tended to be older, with a longer duration of diabetes. There were not significant differences in the prevalence of hypertension, dyslipidemia and obesity (*p >* 0.05). There was early age of diagnosis among case group but not statistically significant (*p =* 0.1).

**Table 1:** The baseline characteristics and diabetic risk factors of type 2 diabetic patients with and without micro-vascular complications

Baseline characteristics	With DR and/or DN *N =* 104	Without DR /DN *N =* 100	*p-*value
Age (years)	58.28 ± 0.60	55.36 ± 0.63	0.001
Sex			
Men	54 (60)	41 (41.0)	0.11
Female	50 (40)	59 (59)	
Duration of diabetes (years)	15.45 ± 0.67	10.21 ± 0.62	0.001
Age of onset (years)	43.22 ± 0.85	45.04 ± 0.75	0.11
Hypertension	76 (73.1)	62 (62.0)	0.09
Dyslipidemia	82 (78.8)	87 (87)	0.12
Obesity	44 (43.1)	37 (37)	0.37
Poor Glycemic control	68 (66)	58(58)	0.23
Albuminuria	53 (51)	-	-
Physical activity			
Vigorous (30 min, at least 5 days per week) Moderate (30 min, 3–4 days per week)	43 (41.3) 16 (15.4)	33 (33) 18 (18)	
Low (10–30 min, less than 3 days per week)	19 (18.3)	19 (19)	0.65
Only daily activity	26 (25)	30 (30)	

Numerical variables were reported as the mean ± standard error, and categorical variables were presented as number (percentage)

Hypertension was defined in subjects with a BP ≥ 140/90 mm Hg or current use of high blood pressure medications. Dyslipidemia was defined as TG >250 mg/dL and/or HDL < 35 mg/dL or using lipid-lowering medications. Poor glycemic control was defined as HbA1c ≥ 7 %. Obesity was classified based on BMI > 30 kg/m2

*DR* Diabetic Retinopathy, *DN* Diabetic Nephropathy

Table 2 shows the means of metabolic markers and all BTMs in serum samples collected from the participants in case and control groups. Compared to the control group, there were no statistically significant differences in FBS, insulin, TC, LDL, Insulin and liver function tests (AST, ALT). However, the case group had significantly lower HDL and higher HbA1c, TG, uric acid, BUN, Cr and eGFR compared with control group (*p <* 0.05).

**Table 2:** The biochemical metabolic and bone turnover markers in diabetic patients who developed microvascular complications compared with those without microvascular complications

Baseline characteristics	With DR and/or DN *N =* 104 (mean ± SE)	Without DR /DN *N =* 100 (mean ± SE)	*p-*value
FBS (mg/dL)	134.81 ± 1.51	134.92 ± 1.03	0.98
Insulin (μU/L)	11.51 ± 0.15	9.08 ± 0.21	0.068
HbA1c (%)	8.08 ± 0.19	7.37 ± 0.14	0.003
TG (mg/dL)	132.55 ± 0.16	115.16 ± 0.15	0.036
HDL (mg/dL)	44.10 ± 1.09	49.58 ± 1.05	0.0001
LDL (mg/dL)	80.19 ± 0.13	79.11 ± 0.13	0.78
TC (mg/dL)	148.91 ± 0.12	148.99 ± 0.12	0.99
Cr (μmol/L)	113.42 ± 0.14	87.51 ± 0.13	0.0001
BUN (mg/dL)	20.59 ± 0.14	15.18 ± 0.13	0.0001
Uric-acid (mg/dL)	5.63 ± 0.15	5.63 ± 0.13	0.002
eGFR	89.02 ± 3.07	76.18 ± 2.70	0.0001
AST (U/L)	17.79 ± 0.23	20.65 ± 0.24	0.56
ALT (U/L)	21.73 ± 0.25	21.73 ± 0.23	0.13
PTH (pg/mL)	46.64 ± 3.91	34.22 ± 1.44	0.001
25(OH)D (nmol/L)	27.24 ± 2.17	33.25 ± 1.99	0.04
OC (ng/mL)	17.88 ± 0.19	13.32 ± 0.16	0.0001
CTX (ng/mL)	0.28 ± 0.02	0.24 ± 0.01	0.06
P1NP (pg/mL)	86.19 ± 0.36	71.52 ± 0.40	0.33

*DN* Diabetic nephropathy, *DR* Diabetic retinopathy**,** *FBS* fasting blood sugar, *TG* triglyceride, *HbA1c* hemoglobin A1c, *HDL* high-density lipoprotein, *LDL* low-density lipoprotein, *TC* total cholesterol, *Cr* creatinine, *BUN* blood urea nitrogen, *AST* aspartate aminotransferase, *ALT* alanine aminotransferase, *PTH* Intact parathyroid hormone, *25(OH)D* 25 (OH) vitamin D, *OC* osteocalcin, *CTX* carboxy-terminal collagen crosslinks, *P1NP* procollagen I aminoterminal propeptide, *eGFR* estimated glomerular filtration

In addition, there was a significant increase trend in serum PTH levels (*p =* 0.0001) and a tendency to be lower in serum levels of 25(OH) D among patients in case group (*p =* 0.04). Also, after adjusting for age, sex and BMI, there was significant difference in serum levels of PTH and 25(OH) D between patients in case and control groups (*p =* 0.005, *p =* 0.042, respectively).

### Bone markers and diabetes microvascular complications

Patients in case group had higher serum levels of P1NP and CTX, although the results were not statistically significant (*p =* 0.33, *p =* 0.058, respectively). However, patients in case group had significantly higher serum levels of OC compared to patients in control group (*p =* 0.0001). The result was consistence even after adjusting for age, BMI, and sex (*p =* 0.001).

**Fig. 1:**
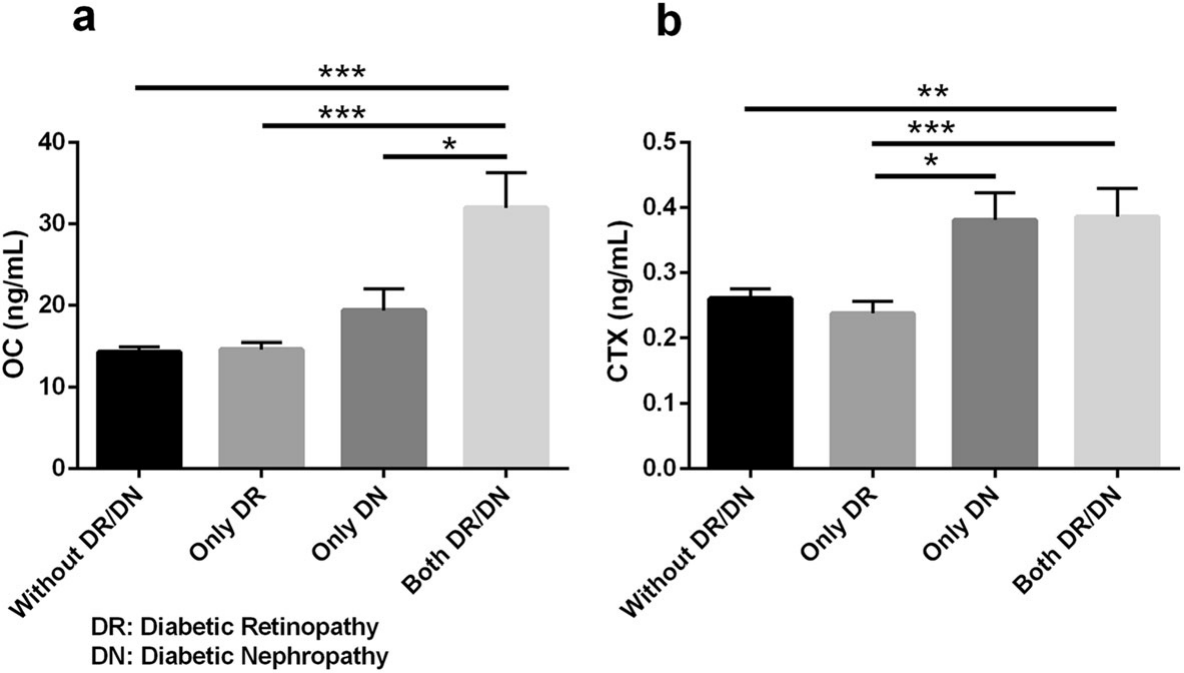
Osteocalcin (**a**) and beta-crosslaps (**b**) in patients with retinopathy (DR), nephropathy (DN) and both complications (DR/DN). Reference group: Type 2 diabetic patients without DR and DN. *p-value < 0.05, **p-value < 0.01, ***p-value < 0.001

To investigate the serum levels of bone markers with respect to type of micro-vascular complication, patients were stratified according to retinopathy or nephropathy or both complications (Fig. 1). The serum levels of OC were higher in patients with both DR/DN compared with patients with only DN or only DR (*p =* 0.0001, *p =* 0.02, respectively) (Fig. 1a).

In addition, in patients with both complications, there were significantly higher serum levels of CTX compared with patients with only retinopathy (*p =* 0.003). But there were not significantly differences in the serum levels of CTX between patients with both complications and those with only nephropathy. Notable, in patients with only nephropathy, the serum levels of CTX were higher than patients with only retinopathy (*p =* 0.025) (Fig. 1b).

### Bone markers and metabolic factors

To investigate correlation between bone markers and metabolic factors, Pearson correlation was performed in all subjects. Among bone markers, there was significant positive correlation between P1NP with ALT levels (r = 0.24, *p =* 0.04) and negative correlation with vitamin D levels (r = −0.18, *p =* 0.01). In addition, there was positive correlation between P1NP and PTH but not significant (r = 0.14, *p =* 0.06). Regarding OC, there were significant positive correlations between OC with Cr (r = 0.55, *p =* 0.0001), BUN (r = 0.57, p0.0001), uric-acid (r = 0.25, *p =* 0.002), PTH (r = 0.53, *p =* 0.0001), and urine albumin/creatinine ratio (r = 0.38, *p =* 0.0001) and negative correlation with eGFR (r = −0.48, *p =* 0.0001).

Significant positive correlations were observed between CTX with PTH levels (r = 0.40, *p =* 0.0001), Cr (r = 0.34, *p =* 0.0001), BUN levels (r = 0.42, *p =* 0.0001) and urine albumin/creatinine ratio (r = 0.23, *p =* 0.001) and negative correlation with eGFR (r = −0.26, *p =* 0.0001). We found no correlation between bone markers and other metabolic factors (*p >* 0.05). The correlations between metabolic factors and bone markers in case and control groups are summarized in Table 3. In case group, there were significant correlations between CTX and OC with kidney function factors (BUN, Cr, eGFR) as well as PTH levels (*p <* 0.01). In control group, there was only significant correlation between OC and CTX with BUN levels (*p <* 0.05).

**Table 3:** Correlation between bone turnover markers and metabolic factors in diabetes patients with and without micro-vascular complications

	With DR and/or DN						Without DR /DN					
CTX (ng/ml)	P1NP (pg/ml)		OC (ng/ml)		CTX (ng/ml)		P1NP (pg/ml)		OC (ng/ml)		CTX (ng/ml)	
Metabolic Factors	r	*p-*value	r	*p-*value	r	*p-*value	r	*p-*value	r	*p-*value	r	*p-*value
FBS (mg/dL)	0.04	0.68	−0.08	0.44	0.01	0.95	0.01	0.92	−0.11	0.33	−0.06	0.60
Insulin ((μU/L)	0.15	0.3	0.22	0.12	0.13	0.37	−0.11	0.37	−0.17	0.17	−0.20	0.10
HbA1c (%)	0.07	0.51	−0.02	0.87	−0.04	0.68	0.11	0.28	−0.13	0.24	−0.04	0.68
TC (mg/dL)	−0.03	0.74	−0.06	0.56	−0.04	0.69	−0.09	0.36	−0.08	0.47	−0.11	0.32
HDL (mg/dL)	0.06	0.58	−0.03	0.75	0.09	0.37	−0.008	0.94	0.08	0.44	0.03	0.79
LDL (mg/dL)	−0.07	0.49	−0.07	0.50	−0.09	0.36	−0.08	0.41	−0.04	0.74	−0.08	0.48
TG (mg/dL)	−0.01	0.92	−0.01	0.93	−0.06	0.57	−0.23^b^	0.03	−0.12	0.29	−0.15	0.17
BUN (mg/dL)	0.09	0.41	0.63^a^	0.0001	0.50^a^	0.0001	0.16	0.12	0.27^b^	0.01	0.21^b^	0.04
Cr ((μmol/L)	0.07	0.5	0.65^a^	0.0001	0.48^a^	0.0001	0.11	0.31	0.15	0.18	0.01	0.95
uric acid	0.11	0.32	0.28^a^	0.006	0.13	0.19	0.04	0.69	0.0001	0.99	−0.07	0.51
eGFR	−0.05	0.61	−0.58^a^	0.0001	−0.40^a^	0.0001	−0.10	0.32	−0.13	0.24	0.03	0.76
Urin-Alb/Cr (mg/g)	−0.09	0.39	0.39^a^	0.0001	0.26^b^	0.01	0.09	0.36	−0.17	0.12	−0.17	0.10
PTH (ng/mL)	0.16	0.13	0.59^a^	0.0001	0.54^a^	0.0001	0.08	0.48	0.17	0.11	0.01	0.95
25(OH)D	−0.17	0.1	−0.06	0.59	−0.11	0.30	−0.18	0.08	−0.01	0.91	−0.02	0.86
AST (mg/dL)	0.14	0.44	.48^a^	0.006	0.38^b^	0.03	0.19	0.22	−0.02	0.88	−0.14	0.38
ALT (mg/dL)	0.29	0.12	0.06	0.74	0.25	0.15	0.26	0.10	−0.19	0.26	−0.31	0.05

*R* Pearson correlation, *DN* Diabetic nephropathy, *DR* Diabetic retinopathy**,** *FBS* fasting blood sugar, *TG* triglyceride, *HbA1c* hemoglobin A1c, *HDL* high-density lipoprotein, *LDL* low-density lipoprotein, *TC* total cholesterol, *Cr* creatinine, *BUN* blood urea nitrogen, *AST* aspartate aminotransferase, *ALT* alanine aminotransferase, *PTH* Intact parathyroid hormone, *25(OH)D* 25 (OH) vitamin D, *OC* osteocalcin, *CTX* carboxy-terminal collagen crosslinks, *P1NP* procollagen I aminoterminal propeptide, *eGFR* estimated glomerular filtration

^a^Correlation is significant at the 0.01 level (2-tailed)

^b^Correlation is significant at the 0.05 level (2-tailed)

A general lineal model was used to control confounding variables. After adjusting for age, sex, obesity and diabetes complications, the analysis showed not significant association between bone markers (OC, CTX, P1NP) and metabolic risk factors including FBS, TG, TC, HDL, LDL, HbA1c, and insulin (*p >* 0.05). However, in this model, there was significant association between OC and CTX with PTH (*p =* 0.0001). Among kidney function markers such as BUN, eGFR, and uric acid; there was significant positive association between OC and CTX with BUN (*p =* 0.0001).

In this model, there was no significant association between bone markers OC and CTX with eFGR (*p =* 0.52, *p =* 0.20, respectively).

We found no significant correlation between serum levels of HbA1c, duration of diabetes with bone turnover markers in case or control groups (*p >* 0.05) even after adjusting age, sex and BMI.

## Discussion

While numerous studies addressed bone markers in T2DM and T1DM [26–28], a few studies compared the difference of bone markers in the context of microvascular complications [29, 30]. Here, we present the difference in bone markers and their association with other metabolic parameters in T2DM with and without microvascular complications.

Our study reveals that bone markers including OC and CTX manifest increased values in T2DM patients with microvascular complications compared with diabetic patients without complications. Along with increased trend in bone markers, we observed increased level of PTH and decreased level of vitamin D in diabetic patients with microvascular complications. Our findings suggest that microvascular complications might involve in disturbance of bone markers and subsequently bone quality in diabetic patients although not provide a direct causality.

In consistent to our study, Inukai and colleges’ evaluated OC, vitamin D and PTH in type 1 diabetes patients with nephropathy. They reported a significant positive correlation between PTH and OC and a positive correlation between OC and urine albumin excretion in diabetes patients. They revealed HbA1C levels were not correlated with OC levels. Also, they found vitamin D levels were decreased and OC levels were increased in diabetic subjects with proliferative retinopathy or with micro- or macro-albuminuria. However, they did not find any significant correlation between OC and vitamin D levels [29]. Our result showed that PTH levels had positive correlation with OC as a bone formation and with CTX as a bone resorption in diabetes patients who suffering of DR/and or DN. Also PTH levels had significant correlation with urine albumin/creatinine ratio. The correlation between circulating PTH and bone markers may modulate bone turnover markers in diabetes patients with micro-vascular complications. It has suggested circulating PTH is important in restoring the reduced OC levels in diabetic patients, probably as a reflection of bone remodeling [29].

A few primary studies, investigated hydroxyproline excretion as a bone resorption markers in type 1 and type 2 diabetes patients with micro-vascular complications [31]. They reported the bone marker did not differ between diabetes patients with or without retinopathy or neuropathy. But there was positive association between hydroxyproline excretion and microalbuminuria.

Interestingly, we could observe robust correlation between bone turnover markers (OC, and CTX) and renal function markers including BUN, uric-acid and eGFR. This association remained significant after adjustment for age, sex and diabetes complications in BUN levels. So, deterioration of renal function in patients with complications which leads to decreased renal clearance of bone markers might at least in part explain the increased value of bone markers in patients with complications.

Our finding also showed no differences in P1NP levels between diabetes patients with or without micro-vascular complications [27]. In contrast to our study, Shanbhogue and colleagues have reported higher level of P1NP in T2DM with or without micro-vascular complications compared to respective controls [26].

Most of studies have reported BTMs in diabetic patients compared to non-diabetic subjects. Generally, they have suggested that in diabetes condition OC and CTX productions were reduced. In a study by Chen and colleagues on type 2 diabetes patients with nephropathy, OC levels were higher than patients without DN. However, compared to healthy subjects, diabetes patients with or without DN had decreased serum OC [2].

Along with increased trend in bone markers, we observed increased level of PTH and decreased level of vitamin D in diabetic patients with microvascular complications. In our study, we did not assess BTMs in healthy group.

Although the patients with complications had higher level of HbA1c and insulin, we could not find any correlation between hyperglycemia markers and bone markers after adjustment for age, sex, obesity and diabetes complications. Conversely, a recent study has indicated the role of OC in replication of beta-cells and insulin secretion and on the other hands role of insulin in activation of OC [32]. The physiologic relevance of this finding has been confirmed by other studies which demonstrated the association between hyperglycemia and bone markers [33, 34]. Nevertheless in agreement with our findings, others have reported no association between bone markers and hyperglycemia. In parallel, another study which examined the effect of a glucose-lowering agents on bone homeostasis in a streptozotocin (STZ)-induced hyperglycemia mouse model, has revealed no alteration in microstructure and bone markers in spite of improving blood glucose [35]. A similar clinical study on newly diagnosed T2DM, has reported that consumption of antihyperglycemic agents had no impact on bone markers while they could improve glucose control in the patients [36]. Our results confirmed the latter studies and suggest that bone turnover markers are not merely influenced by hyperglycemia markers in diabetic patients. Therefore, in the present study, it appears that deleterious effects of micro-vascular complications might not arise from poor hyperglycemia control in these patients.

There are some limitations in our study. The design of our study could not provide a direct causality of diabetes complications and BMTs. However, further studies are needed to investigate the effect of diabetes complications on BMTs and bone status. Consistent with previous studies, our result showed that duration of diabetes significantly differed in patients with and without DR. To control for selection bias due to further match in two groups, we used the univariate model to adjust all confounding variables such as age, sex, and diabetes duration.

## Conclusions

To sum, OC and CTX circulated in higher amounts in T2DM patients with microvascular complications and were not correlated with metabolic markers. In addition, contribution of microvascular complications in bone marker disturbance is likely independent of impaired glucose homeostasis. However, we observed a significant correlation between bone turnover markers and renal function indices. Also PTH levels had significant correlation with CTX and OC as well urine albumin/creatinine ratio. It is likely that circulating PTH is important to keep OC or CTX balance in diabetic patients, probably as a reflection of bone remodeling. Our findings suggest that kidney markers could modulate bone-remodeling in diabetes patients. Little is known on the effect of kidney function on BTM in diabetes patients, further studies are needed.

## Abbreviations

ALT: Alanine aminotransferase
AST: Aspartate aminotransferase
BMI: Body mass index
BTMs: Bone turnover markers
BUN: Blood urea nitrogen
Cr: Creatinine
CTX: Carboxy-terminal collagen crosslinks
CV: Coefficients of variation
DN: Diabetic nephropathy
DR: Diabetic retinopathy
eGFR: Estimated glomerular filtration rate
FBS: Fasting blood sugar
HbA1c: Hemoglobin A1c
HDL: High-density lipoprotein
LDL: Low-density lipoprotein
P1NP: Procollagen I aminoterminal propeptide
PTH: Intact parathyroid hormone
RIA: Radioimmunoassay
T2DM: Type 2 diabetes mellitus
TC: Total cholesterol
TG: Triglyceride
